# Additively Manufactured Ring-Type Thermal Sensor for In-Pipe Flow Monitoring in a Marine Engineering Context: Design Evolution and Electrothermal Characterisation

**DOI:** 10.3390/s26144586

**Published:** 2026-07-20

**Authors:** Dimitrios Nikolaos Pagonis, Christos Liosis, Antonis Vailas, Dimitris Zagklaras, Sotiria Dimitrellou, Eleni Strantzali

**Affiliations:** 1 Naval Architecture Department, University of West Attica, 12243 Athens, Greecesdimitre@uniwa.gr (S.D.); estra@uniwa.gr (E.S.); 2 Mechanical Engineering Department, University of West Attica, 12241 Athens, Greece; liosischristos@uniwa.gr

**Keywords:** additive manufacturing, 3D printing, FDM technology, CNT-PLA, thermal sensor, airflow sensor, COMSOL FEM, hot-ring anemometry, marine industry, in-pipe flow monitoring, on-site fabrication

## Abstract

This work presents the design evolution, fabrication, and characterisation of an additively manufactured ring-type thermal airflow sensor for in-pipe flow monitoring, developed employing exclusively Fused Deposition Modelling (FDM) additive manufacturing technology and a commercially available Carbon Nanotube (CNT)-enriched Biopolymer Polylactic Acid (PLA) composite filament. The design evolution proceeds through three progressive stages. In the first stage, a flat heater element is characterised through Constant-Current (CC) Joule heating experiments in order to derive the corresponding Temperature Coefficient of Resistance (TCR) and Thermal Resistance from the obtained experimental data. Consequently, a Finite Element Method (FEM) model implemented in COMSOL Multiphysics^®^ and calibrated with the extracted material parameters validates the experimental temperature–power relationship and predicts the convective cooling behaviour at various airflow velocities. In the second stage, the geometry is optimised by introducing a conductive trace with a reduced-cross-section central region; as a result, an equivalent thermal localisation is achieved at approximately 26% lower supplied power with respect to the initial heating element, enabled by the design freedom inherent in the FDM process. We should note that the specific sensing geometry can also be directly embedded into any 3D-printed structural component (e.g., a bracket or housing), enabling simultaneous local thermal heating and/or thermal monitoring together with structural functionality within a single printed part. In the third and final stage—the target device—a fully monolithic ring-type airflow sensor is directly integrated into a 3D-printed pipe segment during the printing process. Under constant-current excitation at 40 mA, the device exhibits a monotonically decreasing resistance with increasing airflow (ΔR ≈ 117 Ω over 0–4 m/s) due to convective cooling, while in a single flow-interruption cycle, approximately 79% of the flow-induced resistance change was recovered upon flow removal, with a residual offset of approximately 3% of the heated baseline. A coupled electrothermal FEM model of the device further supports the experimental response by comparing the simulated temperature rise with the values inferred from resistance measurements, while also clarifying the role of the effective internal convective cooling conditions imposed by the pipe geometry. Key features of the proposed device are low raw-consumables cost, fast on-site manufacturing employing a commercially available desktop 3D printer, monolithic construction free of wire-bonded interconnections, and simplicity, indicating its potential for flow monitoring and condition-based maintenance systems aboard vessels as well as in a wide range of industrial sectors. We should note that the present characterisation was performed under laboratory conditions employing a single prototype per design stage; the effects of humidity, salt exposure, vibration, temperature cycling, and material-batch variability remain to be assessed prior to shipboard deployment.

## 1. Introduction

Additive manufacturing (3D printing) is a rapidly evolving process that is continuously emerging into a broad range of industrial and non-industrial applications [1]. Currently, it is widely employed in fields such as aerospace, military, medicine, art, food technology, and many other disciplines [2]; furthermore, additive manufacturing draws considerable attention regarding health and medical-related applications, while its potential to revolutionise the manufacturing industry and transform the production line is broadly accepted. The main reasons supporting this potential are the continuously expanding list of available building materials that can be adopted for manufacturing, the ability to construct complex objects with high dimensional accuracy, and the possibility of on-site manufacturing on demand—all opening new opportunities, particularly in remote operating environments. In addition, by enabling the fast manufacturing of defective replacement parts directly at the point of need, additive manufacturing contributes to longer equipment service life, reduced transport and production costs, a reduced environmental footprint, and an overall contribution to circular economy objectives [3,4].

In the shipbuilding industry, Wärtsilä Corp.—a global leader in smart technologies and complete lifecycle solutions for the marine and energy markets [5]—have been employing additive manufacturing in related manufacturing procedures since mid-2018, including the fabrication of special tools and structural components of engines [6,7]. In more detail, a 3D-printed CE-certified lifting tool was successfully fabricated, able to lift a 240 kg engine piston, saving up to 100,000 euros in tooling costs; the specific case study clearly shows the path of adopting additive manufacturing widely in the maritime sector [7]. Furthermore, the continuously advancing era of artificial intelligence and condition-based maintenance creates a new demand for 3D-printed parts that have sensing capabilities (i.e., custom-made 3D printed sensors), devices that can offer significant advantages compared to their traditional microelectronic counterparts, such as considerably reduced cost, remote and on-demand fabrication, simplicity, durability, and customisation [5]. This direction is supported by recent progress in conductive polymer composites for additive manufacturing, where CNT- and graphene-based thermoplastic nanocomposites have been demonstrated as printable conductive materials for functional components, while recent reviews highlight the broader potential of additively manufactured conductive polymer composites for embedded electrical, sensing, and multifunctional applications [8,9].

It is important to note that the proper operation of all modern vessels relies heavily on the information provided by the onboard measuring devices that quantify critical performance parameters. The typical types of sensors employed are gas-detection sensors, gas/air-flow sensors, humidity sensors, temperature and pressure sensors, speed and acceleration sensors, and strain sensors, all of which are essential with regard to vessels’ safety [10]. Modern sensors that employ semiconductor technology present known drawbacks: a complicated manufacturing process requiring expensive facilities, practically impossible on-site customisation, expensive packaging, and indirect electrical communication between the sensing element and the readout circuitry requiring wire bonding—a delicate process prone to damage, with immediate drawbacks in device complexity, mechanical reliability, process time, and cost [11,12]. Furthermore, maintaining an adequate onboard quantity of spare sensors is very expensive; thus, only sensors required as “critical” spares are stored onboard, in accordance with Section 10.3 of the ISM Code [13]. In case of failure, the ship owner faces the high cost of the sensor and its handling, while on many occasions a waiting period of significant duration must be endured—a constraint that becomes particularly acute when the vessel is in a remote location or when the installed sensor is already obsolete [14].

The main innovation in the current research work is the development of a monolithic, additively manufactured ring-type thermal sensor for in-pipe flow monitoring, through a progressive design evolution employing exclusively FDM technology, commercially available building materials and a standard desktop 3D printer. We should note that the developed devices can be manufactured on-site, on demand, contributing to a reduction in the extent of the required stock-keeping onboard regarding safety-critical equipment and spare parts, in accordance with the relevant regulations [13,14]. The main sensing element of each proposed device is inherently connected to the macroworld, overcoming the necessity for wire bonding, and therefore eliminating the fragile wire-bonded interfaces of conventional counterparts, with expected benefits in device complexity, mechanical reliability, process time, and cost [11,12].

The present work builds upon and extends a series of previous investigations by the authors concerning the development of 3D-printed sensing devices for marine applications [15,16,17]. In reference [15], a vortex shedding flow sensor fabricated exclusively by FDM was presented; the developed device employed a CNT-enriched conductive filament for the piezoresistive sensing element and validated the proof-of-concept for a fully 3D-printed monolithic flow sensor. In reference [16], a 3D-printed strain sensor employing the same CNT-PLA composite material was characterised in detail, revealing the important role of post-printing thermal sintering on the resulting TCR, gauge factor, hysteresis, and linearity of the sensing element. In reference [17], an engine air intake sensor based on additive manufacturing and PCB technology demonstrated the viability of 3D-printed housing geometries—derived through appropriate CFD simulations employing standard NACA airfoil geometries—for mass air flow sensing in marine IC engines, validated on a diesel engine testbed. We should note that thermal anemometry—including hot-wire and hot-film devices—constitutes one of the longest-established approaches for gas-flow and pipe-flow measurement, in which the convective heat exchange between a Joule-heated element and the surrounding fluid provides the sensing signal; the method has been extensively applied both in miniaturised thermal flow sensors [18] and in the study of gas flows in the pipelines of internal combustion engine systems [19]. The present paper addresses a complementary problem: the development of a thermoresistive, Joule-heated element that exploits convective cooling for thermal anemometry, realised as a fully monolithic printed ring embedded within a pipe structure.

In more detail, three progressive design stages are investigated in the present work. The first device (Design 1) is a flat heater element, fully characterised through stepped Joule heating experiments; the extracted TCR and Thermal Resistance values are subsequently employed as material inputs to an appropriate FEM model. The second device (Design 2) features an hourglass-shaped conductive trace, which significantly enhances power efficiency. The third device (Design 3) implements a new sensing concept through a monolithic ring-type sensor embedded into a 3D-printed pipe segment. This “ring-type sensor” is directly integrated into the pipe segment during the printing process, representing a novel concept in thermal sensing device fabrication for flow monitoring applications. We should note that the present work differs from the authors’ previously reported FDM-printed sensing devices in three respects: in contrast to the vortex shedding flow sensor [15], the piezoresistive strain sensor [16], and the engine air intake sensor [17], which constitute discrete devices subsequently mounted to the measurement site, the sensing element here is co-printed monolithically within the wall of the flow conduit itself in a single print job; the transduction relies on the steady-state thermoresistive response of a Joule-heated element to convective cooling, rather than on vortex shedding [15] or strain [16]; and, while the thermal anemometric principle is shared with [17], the ring geometry provides symmetric, non-intrusive exposure across the full pipe bore, and the development is supported throughout by a coupled electrothermal FEM methodology whose material inputs are transferred and validated across the successive design stages. The fabricated device was experimentally evaluated under varying airflow conditions at constant-current excitation of 40 mA to assess its thermal response under different airflow velocities (0 to 4 m/s).

In order to allow an objective assessment of the outcome, the following quantitative targets were defined: (i) for the ring-type sensor, a monotonic resistance response over the 0–4 m/s range with a total resistance variation of at least 10% of the heated no-flow baseline; (ii) for the intermediate design stage, a reduction in the supplied power of at least 20% for equivalent peak temperature, achieved through geometry alone; and (iii) agreement between the FEM-predicted and the experimentally estimated temperature rise within approximately 10% for the zero-flow and the developed forced-flow conditions. We should note that the specific threshold values were selected as follows: the 10% full-span resistance variation ensures that the flow-induced signal exceeds both the resistance-measurement uncertainty and the observed plateau scatter (each below 0.4% of the corresponding resistance level; see Section 2.5 and Section 5.3) by more than an order of magnitude; the 20% power reduction corresponds to a level of improvement that is clearly distinguishable from measurement uncertainty and of practical significance for the power-budget constraints of battery-operated or remotely powered sensing systems; and the approximately 10% FEM-agreement figure reflects the expanded uncertainty of the experimentally estimated temperature rise (approximately 11% at a coverage factor k = 2; see Section 5.5), since demonstrating agreement tighter than the experimental uncertainty itself would not be meaningful. The specific targets are revisited in Section 6.

The remainder of the paper is organised as follows: Section 2 describes the materials, fabrication, experimental setup, and finite-element modelling approach; Section 3 presents the electrothermal characterisation and FEM convective-cooling prediction for Design 1; Section 4 discusses the Design 2 geometry and its improved thermal localisation and power efficiency; Section 5 presents the monolithic ring-type sensor, its constant-current flow response, signal reversibility, and coupled electrothermal FEM analysis; Section 6 provides a discussion while Section 7 states the conclusions.

## 2. Materials and Methods

### 2.1. Conductive Composite Filament

The conductive sensing elements for all three design stages were fabricated from the commercially available CNT-enriched PLA composite filament FiberForce Nylforce CNT Conductive (FiberForce Co., Treviso, Italy) [20]. According to the manufacturer, the specific material consists of a biopolymer compound enriched with Carbon Nanotubes (CNTs); its nominal surface electric resistivity is 10 Ω/sq [20]. We should note that the specific filament was selected due to its noticeably higher conductivity compared to other commercially available conductive filaments, as already reported in [15], where the same material was employed for the piezoresistive sensing element of a vortex shedding sensor. Furthermore, the same filament was employed in the strain sensor developed in [16], where its thermoresistive and piezoresistive properties were characterised in detail. The non-conductive structural elements were fabricated from standard Polylactic Acid (PLA) filament (UltiMaker, Utrecht, The Netherlands). The effective material properties adopted in the FEM model for both filaments are presented in Table 1. The values for standard PLA are based on the manufacturer’s specifications [21] and the literature, adjusted for the effects of the FDM printing process and post-printing thermal treatment, as discussed together with the conductive CNT filament properties in Section 2.4.

### 2.2. Device Fabrication

All three designs were fabricated through standard FDM technology employing the commercially available desktop Ultimaker S3 printer (UltiMaker, Utrecht, The Netherlands) [21]; according to the manufacturer’s specifications, the printer offers a minimum layer height of 20 μm (depending on nozzle size and print settings), while its nominal XYZ resolution is 6.9 μm, 6.9 μm, and 2.5 μm for each axis, respectively. The dual-extrusion capability of the specific printer enables simultaneous deposition of the conductive CNT-PLA and the non-conductive standard PLA in a single print job, eliminating post-processing assembly steps and ensuring reliable adhesion between the conductive and insulating regions of each device. All devices were printed using 2.85 mm filament for both materials and 0.4 mm nozzles for both print cores, with a layer height of 0.2 mm and a bed temperature of 70 °C. The standard PLA was deposited with Nozzle 1 at 190 °C, with a standby temperature of 150 °C and a print speed of 40 mm/s, whereas the conductive CNT-PLA was deposited with Nozzle 2 at 250 °C, with a standby temperature of 230 °C and a print speed of 10 mm/s. The infill density was set to nominally 100% using a concentric infill pattern, while the perimeter-shell number and the horizontal and vertical shell thicknesses were set to zero. Therefore, the printed conductive and insulating regions were defined primarily by the slicer-generated concentric infill paths (Ultimaker Cura version 4.11; UltiMaker, Utrecht, The Netherlands), rather than by separate outer shell contours. The same material-specific printing parameter set was kept identical across all three designs, since the deposition conditions and toolpath pattern of the conductive filament are known to affect the connectivity of the CNT network and, consequently, the electrical and thermal transport properties of the printed element. We should note that, in agreement with the previous findings [15,16] for the same conductive CNT-PLA material, a post-printing thermal treatment at a temperature of 100 °C for 24 h was applied to all the sensing elements. As reported in [15], a significant total decrease of approximately 28% of the initial resistance value was obtained after performing twelve steps of two-hour thermal sintering at the specific temperature, attributed to the enhancement of conductive pathways within the CNT network, while a similar effect was observed in [16] and confirmed in the present case. The specific treatment was performed in a laboratory convection oven in ambient air (of approximately 27 °C) with heating to the 100 °C set point at approximately 2 °C/min, followed by unforced cooling to room temperature inside the oven; no measurable dimensional change in the printed structures was observed after treatment, the effect on the sensing elements being confined to the reduction in resistance noted above. Electrical connections to the readout circuit were established via conductive adhesive on top of the integrated conductive pads (Design 1) or injected into dedicated side holes in the conductive pads (Designs 2 and 3), the latter approach yielding improved contact reliability.

### 2.3. Experimental Setup

**Figure 1:**
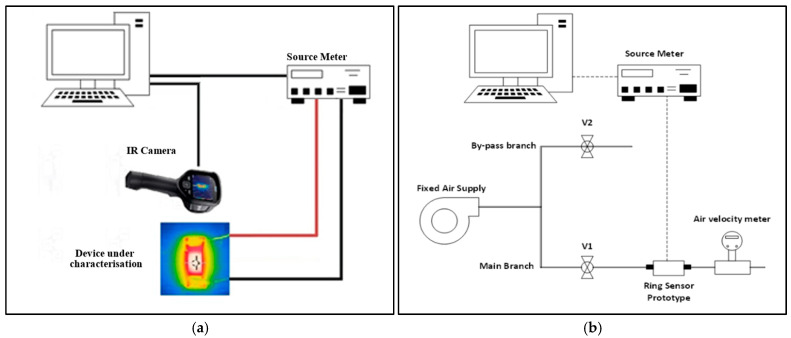
Experimental setups employed: (**a**) electrothermal characterisation of Designs 1 and 2; (**b**) flow characterisation of the pipe-integrated ring-type sensor.

Electrical characterisation of all devices was performed employing a Keithley 2401 (Solon, OH, USA) source meter, which applied stepped constant-current excitation to the device under test while simultaneously measuring the terminal voltage; the resistance at each step was deduced as R = V/I. For Designs 1 and 2, stepped constant-current excitation from 5 to 80 mA in 5 mA increments (sixteen non-zero current levels) was applied starting from the unpowered state, with a stabilisation delay of 2.5 s per step. The unpowered condition served only as the ambient starting state and was excluded from all subsequent analysis. During these measurements, thermal imaging was performed employing a FLIR E54 infrared camera (FLIR Systems, Wilsonville, OR, USA), providing real-time two-dimensional temperature maps at each current step; the maximum temperature at each step corresponds to the peak pixel value extracted from the thermal image recorded at the end of the corresponding step. An emissivity setting of 0.95 was used for both the CNT-PLA and PLA printed surfaces; this value is commonly adopted for matte, non-metallic surfaces and was considered appropriate for the printed polymer surfaces examined here. The camera was positioned approximately 30 cm above the device under test, while the reflected apparent temperature was set equal to the measured ambient laboratory temperature of approximately 300 K. We should note that the peak-pixel temperature is sensitive to the assigned emissivity and to image noise; however, since the examined surfaces are matte polymeric surfaces with high emissivity, the associated contribution was treated within the practical IR-temperature uncertainty discussed in Section 2.5 and propagated to the TCR uncertainty in Section 3.2. Furthermore, in contrast to the considerably low excitation current that was employed during the piezoresistive characterisation in [16]—in order to avoid self-heating effects arising from the high TCR of the CNT composite—the opposite approach was selected for the thermal devices characterisation, since Joule self-heating constitutes the operating mechanism; thus, the applied current values were selected to produce a measurable temperature rise at the sensing element. The experimental setup employed for the electrothermal characterisation is presented in Figure 1a.

For the flow characterisation of the ring-type sensor (Design 3), the experimental setup presented in Figure 1b was employed; it mainly consists of an industrial centrifugal fan with a fixed air supply, an air velocity measuring unit, an appropriate flow network, two needle valves and a Keithley 2401 source meter. The flow network comprises two branches, the main one where the sensor under characterisation is mounted and a second one which acts as a bypass for reducing the incoming flow from the centrifugal fan. The value of the desired flow is set by adjusting the needle valve (V1), which is situated before the prototype sensor and the bypass section valve (V2) of the setup, while the resulting air velocity is monitored continuously by the air velocity measuring unit positioned in open air immediately downstream of the sensor-assembly outlet. In more detail, the reported air-velocity values correspond to the reading of the vane-type measuring unit (UNI-T UT363, Uni-Trend Technology, Dongguan, China) placed in open air immediately downstream of the sensor-assembly outlet. Since the measurement is performed at a small distance from the sensor-assembly outlet, where the emerging airflow has not yet undergone significant expansion or mixing, the measured velocity was used as the reference velocity of the specific flow configuration and as a representative value of the mean air velocity through the 21 mm sensor bore, rather than as a centreline or local point velocity. We should note that, at the specific measurement position, the vane reading corresponds closely to the mean velocity of the airflow leaving the 21 mm bore, within the instrument accuracy discussed in Section 2.5; the abrupt inlet contraction and any associated local recirculation influence the local velocity profile and the convective heat transfer at the ring element—the latter being addressed through the correction factor discussed in Section 5.5—but not the volumetric flow rate itself, which is fixed by continuity. Verification of the velocity assignment against a traceable reference is identified in the future work of Section 7. For each imposed flow condition, the vane-anemometer readings were manually logged after stabilisation and averaged to obtain the reported velocity value. We should note that the specific experimental setup is identical to the one employed in [15] for the characterisation of the vortex shedding flow sensor. Constant-current operation at 40 mA was employed, while the corresponding resistance of the heated element was monitored as a function of the applied airflow; the air velocity range employed in the characterisation of Design 3 is from 0 to 4 m/s.

### 2.4. Finite Element Modelling

FEM simulations were performed in COMSOL Multiphysics^®^ (version 5.6, COMSOL AB, Stockholm, Sweden) coupling the Electric Currents and Heat Transfer in Solids interfaces. The material properties summarised in Table 1 were assigned to the conductive PLA domain, with electrical resistivity modelled as temperature-dependent according to:

ρ(T) = ρ_0_ · [1 + α · (T − T_0_)] (1)

where ρ_0_ is the reference resistivity, α is the Thermal Coefficient of Resistance (TCR), and T_0_ is the reference (environment) temperature. Convective heat transfer at the exposed surfaces was modelled using a convective heat flux boundary condition with a fixed heat transfer coefficient, h. For Design 1, the h values were derived analytically employing the flat-plate Nusselt number correlation for laminar external flow Equations (2) and (3):

Nu = 0.664 · Re^½^ · Pr^⅓^ (2)

h = Nu · k_air_/L (3)

where the Reynolds number Re is defined as:

Re = ρ_air_ · U · L/μ_air_ (4)

In the above equations, the values of Prandtl number (Pr), thermal conductivity (k_air_), density (ρ_air_) and the dynamic viscosity of air (μ_air_) are equal to 0.71, 0.02638 W/(m·K), 1.18 kg/m^3^, and 18.45 × 10^−6^ Pa·s, respectively, assuming a temperature of 300 K; the characteristic length of the heater (L) is 5 mm. Note that for the air-velocity range considered, the corresponding Reynolds number remained below 4.0 × 10^3^, i.e., well below the typical transition threshold for external flow over a flat plate, thereby supporting the laminar-flow assumption. Rather than employing a full CFD solver, the above analytical approach was selected for calculating h; this is consistent with the simplified heat transfer analysis employed in [17] for estimating convective heat transfer over the hot-wire sensing element, where the same empirical equation was employed to establish the expected saturation behaviour of the sensor signal. The derived h values for the air velocity range considered are presented in Table 2.

For Design 3, where the heated ring element is situated inside a pipe of inner diameter D = 21 mm, internal pipe flow conditions apply. Thus, the Reynolds number (Re_pipe_) is defined as:

Re_pipe_ = ρ_air_ · U · D/μ_air_ (5)

For the air velocity range employed in the characterisation (0–4 m/s), the corresponding Reynolds number exceeds the transition threshold (Re_pipe_ > 2300) at velocities of approximately 2 m/s and above, indicating transitional-to-turbulent flow. For the laminar flow range (Re_pipe_ < 2300), a constant Nusselt number Nu = 4.36 was adopted, corresponding to fully developed internal flow under constant heat flux conditions; the specific boundary condition is appropriate since the ring element operates under constant-current Joule heating. For the transitional and turbulent portion (Re_pipe_ > 2300), the Dittus–Boelter correlation Equation (6) for fully developed turbulent internal flow was employed. We should note that the specific correlation is strictly valid for Re > 10,000; it is applied here as a first-order approximation since Re_pipe_ reaches approximately 5400 at the maximum tested velocity. Thus:

Nu__D_ = 0.023 · Re_pipe_^0.8^ · Pr^0.4^ (6)

h = Nu__D_ · k_air_/D (7)

where Re_pipe_ is based on the pipe inner diameter D, and the exponent 0.4 corresponds to heating of the fluid (the internal ring surface is hotter than the incoming air). The derived h values for Design 3 are presented in Table 2 together with the estimated ones for Design 1. For zero airflow velocity, natural convection was assumed on all surfaces, i.e., h = 8 W/(m^2^·K) for surfaces exposed to the open laboratory environment (Design 1 and Design 3 external surfaces) and h = 3 W/(m^2^·K) for the internal surfaces of the Design 3 tube segment, reflecting the reduced convective circulation within the pipe.

We should note that for both building materials, CNT-enriched conductive PLA and standard PLA, the physical properties employed in the FEM model (summarised in Table 1) represent effective values calibrated to the printed structure. In more detail, a density of 1240 kg/m^3^ was adopted for the CNT-enriched material, corresponding to an approximately 8% reduction with respect to the manufacturer’s nominal bulk value of 1350 kg/m^3^ [20]; the specific reduction accounts for the printing-induced porosity and inter-bead micro-voids inherent to FDM structures, arising from incomplete neck growth between adjacent deposited filaments even at nominal 100% infill, and reported to reduce the density of FDM-printed parts by more than 8% with respect to the raw feedstock [22]. The same porosity reduction was applied to the standard PLA domain, yielding an effective printed density of 1141 kg/m^3^ (reduced from the manufacturer’s nominal value of 1240 kg/m^3^ [21]). Similarly, the thermal conductivity adopted for the standard PLA material, equal to 0.26 W/(m·K), is higher than the typical value for amorphous PLA (approximately 0.15 W/(m·K)); the increase is attributed to the enhanced crystallinity resulting from the post-printing thermal treatment described in Section 2.2, consistent with reported values for thermally processed 3D-printed PLA [23].

The electrical resistivity of the standard PLA domain was set to 1.78 × 10^8^ Ω·m, based on the bulk conductivity reported by Wei et al. [24] for unfilled PLA (σ = 5.61 × 10^−9^ S/m); this confirms the effectively insulating character of the PLA substrate relative to the conductive CNT-enriched element. Regarding thermal conductivity of the CNT-enriched material, an effective value of k = 4.5 W/(m·K) was assigned; this value is noticeably higher than that of pure PLA, reflecting the contribution of the CNT network to in-plane heat conduction within the printed element. We should note that the choice of k = 4.5 W/(m·K) is supported by the simplified one-dimensional thermal resistance analysis presented in Section 3.3, which yields an upper-bound estimate of approximately 5.1 W/(m·K) for the effective conductivity of the central heating region.

Furthermore, the model incorporates the experimentally derived TCR and bulk reference resistivity (ρ_0_, at room temperature) values of 0.0075 K^−1^ and 0.204 Ω·m, respectively, the derivation of which is presented in Section 3.2. Finally, the specific heat capacity was retained equal to that of standard PLA (1200 J/(kg·K)) [25], since the effect of the CNT filler on C_p_ at the specific loading is negligible.

### 2.5. Measurement Uncertainty and Scope of the Characterisation

The resistance values reported throughout this work were deduced as R = V/I from the source meter readings (Keithley 2401); according to the manufacturer’s specifications, the corresponding accuracy is ±(0.034% + 20 μA) for the sourced current (100 mA range) and ±(0.012% + 2.4 mV) for the measured voltage. Treating the voltage and current uncertainty contributions as independent and combining them in quadrature, as appropriate for the first-order propagation of products or quotients of measured quantities, the resulting relative resistance uncertainty at the 40 mA operating point is below 0.1%, i.e., approximately 0.5 Ω at the 500 Ω level. The steady-state fluctuation of the resistance signal during the flow characterisation presented in Section 5 was quantified as the sample standard deviation over the final forty samples, corresponding to approximately the last eight minutes, of each velocity plateau. The resulting values are reported alongside the flow characterisation in Section 5.3. We should note that the manufacturer figures above represent accuracy limits rather than a direct estimate of the random noise in the resistance time series; therefore, the observed plateau scatter should not be interpreted as source-meter noise alone, but as the combined scatter of the complete measurement system during the flow characterisation.

Surface temperatures were recorded with a FLIR E54 infrared camera, with a specified accuracy of ±2 °C or ±2% of reading, whichever is greater. Thus, regarding the propagation of this accuracy to deducing TCR, two contributions were considered. A common temperature-offset error would mainly shift the R–T characteristic along the temperature axis and would not significantly affect the fitted slope, whereas a proportional temperature-scale error rescales the temperature axis and therefore propagates directly to the slope. Since the TCR is deduced from the fitted slope, α = (dR/dT)/R_0_, the proportional component of the camera accuracy was taken as the relevant first-order temperature contribution to the TCR uncertainty. The second contribution is the statistical uncertainty of the fitted R–T slope itself, evaluated from the regression as the relative standard error of the fitted slope, SE(b)/b. The numerical evaluation of the TCR uncertainty is reported alongside the R–T characterisation in Section 3.2.

Consequently, the experimentally estimated temperature-rise values employed in Section 5.5, obtained as ΔT = (R − R_ref_)/(R_ref_ · α), where R_ref_ is the corresponding ambient temperature reference resistance of the active sensing element, inherit the TCR uncertainty as their dominant contribution. Resistance scale errors are largely common to R and R_ref_, since both quantities are measured with the same instrument and on the same range; their influence on the calculated temperature rise is therefore reduced compared with the uncertainty associated with the TCR. The remaining uncertainty contribution arises from the random scatter of the plateau resistance values, which is evaluated from the sample standard deviations reported alongside the flow characterisation in Section 5.3. In more detail, since the experimentally estimated temperature rise is proportional to the resistance increase from the ambient temperature reference, ΔT∝ $\bar{R}$_plateau_ − R_ref_, where $\bar{R}$_plateau_ is the mean resistance of each velocity plateau, the uncertainty contribution associated with plateau resistance scatter is expressed in relative form as $\sigma_{R}$/($\bar{R}$_plateau_ − R_ref_). The numerical uncertainty assigned to the experimentally estimated ΔT values is reported in Section 5.5, where the FEM–experiment comparison is performed.

The airflow velocity was monitored employing a handheld vane anemometer (UNI-T UT363) with a specified accuracy of ±5% of reading and a resolution of 0.1 m/s, corresponding to a velocity uncertainty between approximately ±0.08 m/s and ±0.21 m/s over the examined range.

We should note that a single prototype was fabricated and characterised for each design stage, in line with the proof-of-concept character of the present design-evolution study; accordingly, the reported dependences quantify the repeatability of the measurements performed on each device rather than device-to-device reproducibility. In this respect, the TCR deduced in Section 3.2 is consistent with the pronounced thermoresistive sensitivity reported in [16] for the same filament, obtained on an independently printed device of different geometry, while the transfer of the deduced value to the ring geometry of Design 3 (Section 5.5) provides a first indication of cross-device consistency of the extracted effective parameters; the fabrication and characterisation of multiple nominally identical prototypes for establishing full device-to-device statistics is identified as future work in Section 7.

## 3. Design 1: Initial Flat Heater—Characterisation and FEM Convective Cooling Prediction

### 3.1. Device Geometry

**Figure 2:**
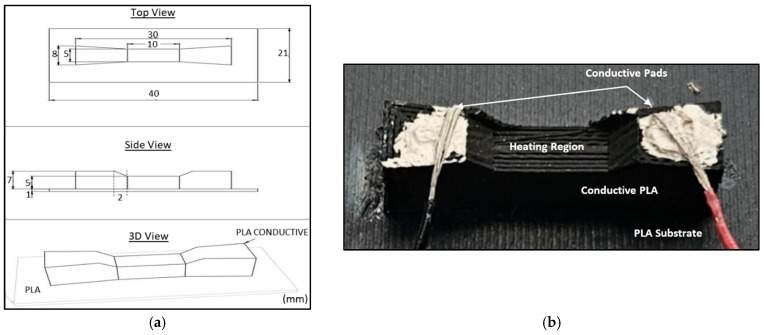
Design 1—Initial flat heater: (**a**) engineering drawing (top, side, and 3D views); (**b**) photograph of the fabricated prototype with integrated electrical contacts.

The first device generation consists of a flat heater element with a CNT-PLA conductive core co-printed within a non-conductive PLA substrate. The overall footprint is 40 × 21 mm, with a 1 mm thick base plate and a total assembly height of 8 mm. The conductive element has a tapered geometry in both width and height; it has a total length of approximately 30 mm, comprising a central heating region 10 mm in length and 5 mm in height, which transitions to wider and taller pads at each end, with dimensions of 8 mm × 7 mm. Electrical connections are established via conductive adhesive to the integrated conductive pads. The geometry is presented in Figure 2 together with a photograph of the fabricated device.

### 3.2. TCR and Electrical Characterisation

**Figure 3:**
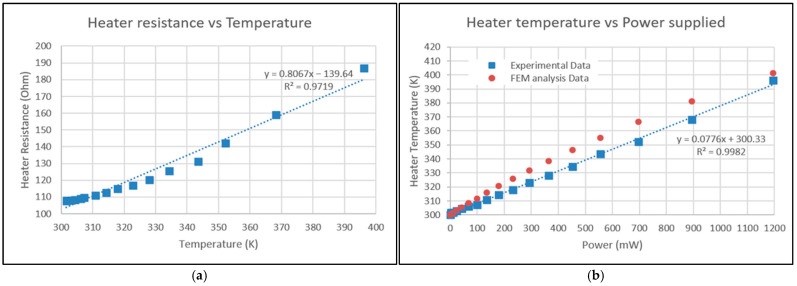
Design 1 electrothermal characterisation: (**a**) heater resistance as a function of heater temperature; (**b**) heater temperature as a function of supplied power, with corresponding FEM results shown for comparison.

The device was characterised by applying constant-current steps from 0 to 80 mA in 5 mA increments (sixteen non-zero levels), with a stabilisation delay of 2.5 s per step; at the end of each step, the resistance (R_h_) and the corresponding maximum surface temperature (T_h_) extracted from the IR thermal image were recorded simultaneously. We should note that, since R_h_ and T_h_ are sampled simultaneously and therefore refer to the same instantaneous thermal state of the element, the validity of the R–T pairing employed for the TCR derivation does not require the attainment of full thermal steady state at each step. The measured R–T relationship is presented in Figure 3a; an approximately linear trend is observed across the measured range (300–396 K), consistent with the positive TCR behaviour of CNT-enriched thermoplastic composites, as reported in [16] for the same conductive filament. A linear regression on the specific data yields:

R_h_(T) = 0.8067 · T_h_ − 139.64,  R^2^ = 0.9719 (8)

The TCR is accordingly derived as α = (dR/dT)/R_0_ = 0.8067/107.68 ≈ 0.0075 K^−1^, where R_0_ = 107.68 Ω is the reference resistance at T_0_ ≈ 300 K, measured at the lowest current step (5 mA), where self-heating is negligible. We should note that the measured resistance corresponds to the entire conductive path, including the tapered transition regions, whereas the temperature is extracted as the peak pixel value of a spatially non-uniform, self-heated element. Therefore, the deduced TCR represents an effective device-level TCR of the printed element under the specific measurement convention, rather than an intrinsic material property of the composite. Note that the same convention is retained when the specific value is subsequently employed in the FEM models of Section 3.4 and Section 5.5, i.e., the parameter is applied self-consistently throughout the present work, while the transfer of the specific value to the independently printed ring geometry of Design 3 reproduces the zero-flow temperature rise within approximately 3% (Section 5.5). Thus, the specific effective value can be regarded as transferable across devices fabricated from the same material and process.

Applying the uncertainty procedure described in Section 2.5, the statistical contribution to the TCR uncertainty is estimated from the regression as SE(b)/b = [(1/R^2^ − 1)/(*n* − 2)]^1/2^ ≈ 4.5% for R^2^ = 0.9719 and *n* = 16 current steps. Combining this regression contribution with the ±2% proportional temperature-scale contribution of the IR camera in quadrature yields a relative uncertainty of approximately ±5% for the deduced TCR, dominated by the regression scatter. We should note that the specific characterisation corresponds to a single printed device and a single stepped current sweep of sixteen steps, consistent with the scope defined in Section 2.5. The earlier work cited in [16] provides relevant context by noting that CNT-enriched thermoplastic composites can exhibit high TCR magnitudes, with values as large as −1.28 × 10^−2^ °C^−1^ reported in the literature; full device-to-device statistics across samples, printers, and filament batches remain part of the future work of Section 7. It should also be noted that the sign and magnitude of TCR in CNT/polymer composites are sensitive to filler concentration, matrix properties, and processing conditions, with both positive and negative values reported in the literature at comparable loadings [26,27]; the present characterisation reports a positive TCR of 0.0075 K^−1^ for the specific CNT-PLA filament. This observation is also consistent with recent work on three-dimensionally printed temperature sensors based on commercial conductive PLA materials, where resistance variation with temperature was exploited for thermal monitoring in additively manufactured structures [28].

The bulk resistivity was not estimated by attributing the full measured resistance solely to the 10 mm central heating region, since the conductive path also includes the two tapered transition regions adjacent to the conductive adhesive contacts, as presented in Figure 2a; as a first-order correction, the conductive path can be represented by three resistors in series: the 10 mm central region and the two 2 mm tapered regions. Since the central cross-sectional area A_c_ = 5 × 5 = 25 mm^2^, the resistance of the central region is:

R_c_ = ρ · (10/25) = 0.4 · ρ (9)

where, for convenience, in the following geometric calculations, ρ is temporarily expressed in Ω·mm. For each tapered cross-sectional area, the width w(x) increases linearly from 5 to 5.6 mm over the inner 2 mm, while the height h(x) increases linearly from 5 to 7 mm; therefore:

w(x) = 5 + 0.3 · x(10)

h(x) = 5 + x(11)

where 0 ≤ x ≤ 2 mm. Thus, the local cross-sectional area in the tapered region is:

A(x) = (5 + 0.3 · x) · (5 + x)(12)

and the resistance R_t_ of one tapered region is equal to:

(13) $$R_{t}=\rho \cdot \int_{0}^{2}\frac{dx}{\left(5+0.3\cdot x)\cdot (5+x\right)}\approx 0.06376\cdot \rho$$

Hence, the total resistance of the device becomes R = R_c_ + 2 · R_t_ = 0.5275 · ρ. Employing the measured value for the room-temperature resistance R_0_ = 107.68 Ω, the deduced bulk resistivity becomes ρ_0_ = R_0/_(0.5275 × 10^3^) = 0.204 Ω·m.

### 3.3. Thermal Resistance

The relationship between supplied power (P) and heater temperature (T_h_) is presented in Figure 3b; a high degree of linearity is observed across the full range of applied power since a linear regression on the specific data yields:

T_h_(P) = 0.0776 · P + 300.33,  R^2^ = 0.9982 (14)

The slope of the above relationship yields a Thermal Resistance of Rth = ΔT/P ≈ 78 K/W. The high linearity (R^2^ = 0.9982) is consistent with conduction through the PLA substrate being the dominant heat dissipation mechanism at the power levels considered, which is in accordance with the expected behaviour for a compact PLA element at moderate power input. The physical origin of the observed linearity lies in the conduction-dominated character of the heat dissipation; that is, as long as the effective thermal conductivity of the printed structure remains essentially constant over the examined range, the Thermal Resistance is temperature-independent and the T–P relationship remains linear. The competing nonlinear mechanisms are weak in the specific range, since radiative losses contribute only a small fraction of the total dissipation at the examined temperatures, while the temperature dependence of the natural-convection coefficient over 300–396 K is modest. Similarly, the linearity of the R–T characteristic in Figure 3a reflects the operation of the percolated CNT network in a regime where the inter-particle conduction paths are not sufficiently disturbed to destroy the approximately linear response. The post-printing thermal treatment applied during fabrication (Section 2.2) has been shown in [16] to stabilise the conductive network of the specific filament, and the same mechanism is expected to limit aging effects upon repeated heating and cooling cycles. It should be acknowledged, however, that possible residual first-cycle settling of the conductive network cannot be excluded and may contribute to small baseline shifts during subsequent thermal-flow cycling; therefore, the systematic verification of long-term stability under repeated thermal cycling is included in the future work of Section 7. We should note that the 2.5 s per-step delay employed in the stepped characterisation cannot in itself guarantee complete thermal equilibration of the printed element at every step; the protocol should therefore be regarded as quasi-steady. Nevertheless, the consistency of the deduced slope with the steady-state FEM solution of Section 3.4, together with the close zero-flow agreement obtained in Section 5.5 when the extracted parameters are transferred to the ring geometry, indicates that any residual departure from full steady state has a limited effect on the derived Thermal Resistance value.

Note that the derived TCR (α = 0.0075 K^−1^) and resistivity (ρ_0_ = 0.204 Ω·m) were subsequently adopted as the material inputs to the FEM model. Additionally, as presented in Table 1, the value of thermal conductivity (k) of 4.5 W/(m·K) for the conductive CNT-PLA was selected as an effective parameter for the FEM model, as it provides reasonable agreement with the experimentally observed thermal resistance and temperature–power response of Design 1. It should be noted that, for a simplified one-dimensional approximation taking into consideration only the central heating region, the effective thermal conductivity can be estimated as k = L/(A·R_Th_), where (L) and (A) correspond to the characteristic length and cross-sectional area of the central area, respectively. This simplified estimation yields an upper-bound effective conductivity of approximately 5.1 W/(m·K); the FEM value of k = 4.5 W/(m·K) is therefore in good agreement with the 1D upper-bound estimate. The difference is attributed to convective and radiative losses at the exposed surfaces, which are absent from the simplified 1D estimate. We should note that this attribution can be quantitatively constrained from quantities already employed in the present work. The heated central element measures approximately 10 × 5 × 5 mm; since its base rests on the printed substrate and its two end faces feed into the contact pads, the exposed surface comprises the top and the two lateral faces, i.e., an exposed area A ≈ 150 mm^2^ (1.5 × 10^−4^ m^2^). Taking the temperature rise in the element at the highest current step from the electrothermal characterisation performed for the TCR in Section 3.2, ΔT ≈ 96 K above the ambient value, the natural-convection loss (Q_conv_ = h·A·ΔT) evaluates to approximately 0.12 W, where h = 8 W/(m^2^·K) is the representative natural-convection coefficient (the value subsequently adopted for the externally exposed surfaces of the ring-type sensor FEM model, Section 5.5). The corresponding radiative loss (Q_rad_ = ε·σ·A·(T_s_^4^ − T_amb_^4^)) evaluates to approximately 0.13 W, where ε = 0.95 is the surface emissivity adopted for the printed polymer surfaces (Section 2.3), σ is the Stefan–Boltzmann constant (σ = 5.67 × 10^−8^ W/(m^2^·K^4^)), T_s_ ≈ 396 K is the surface temperature and T_amb_ ≈ 300 K is the ambient temperature. Their sum, approximately 0.25 W, corresponds to about 20% of the supplied power of approximately 1.2 W at the specific step; thus, no less than approximately 80% of the supplied power is dissipated by conduction into the printed substrate and the contact regions, consistent with the conduction-dominated interpretation above. It should be noted that this conclusion is not sensitive to the adopted natural-convection coefficient, since even doubling its value leaves the combined convective and radiative share below one third of the supplied power.

### 3.4. FEM Validation and Convective Cooling Prediction

The previously extracted effective electrothermal parameters of the printed CNT-PLA heater (TCR and reference resistivity) were employed to parameterise the corresponding COMSOL model, which was subsequently validated against the experimental T–P data; simulated T_h_ values at the same power steps are overlaid in Figure 3b, showing reasonably good agreement, particularly at moderate power levels (approximately below 300 mW). Note that the simulation slightly overestimates the maximum temperature reached at higher power inputs. This deviation is attributed primarily to simplified boundary conditions and constant-property assumptions, which do not fully capture parasitic heat losses (e.g., through electrical contacts and wiring) or the non-uniform thermal response of the printed CNT-PLA/PLA structure, arising from its 3D-printed morphology (e.g., internal microvoids). In addition, at temperatures near or above the glass transition region of PLA, the thermally induced softening of the PLA matrix may alter the CNT conductive network and thus the effective thermal and electrical response of the composite, further contributing to the recorded deviation. Such effects are not captured by the constant-property material model employed. In addition, surface-to-ambient radiation was not included as a separate boundary condition in the FEM analysis of the present work; its potential magnitude can be assessed through the linearised radiative heat transfer coefficient, h_rad_ = ε·σ·(T_s_ + T_amb_)·(T_s_^2^ + T_amb_^2^), which evaluates to approximately 9 W/(m^2^·K) at the highest examined surface temperature (T_s_ ≈ 396 K, ε = 0.95) and decreases to approximately 6 W/(m^2^·K) near ambient temperature. Therefore, radiation acts as an additional surface-loss path of magnitude comparable to natural convection across the entire examined temperature range, consistent with the estimate presented in Section 3.3. We should note that its omission acts in the direction of the residual deviation observed in Figure 3b, since including the radiative loss would reduce the predicted temperature at the highest power levels. For the forced-flow cases of the ring-type sensor, discussed in Section 5.5, the internal heat-transfer level is calibrated against the experimental data; thus, any radiative contribution is implicitly absorbed by the specific correction factor, while at zero flow the total effective conductance is dominated by conduction into the printed pipe structure, limiting the influence of the omitted radiative term on the corresponding prediction.

**Figure 4:**
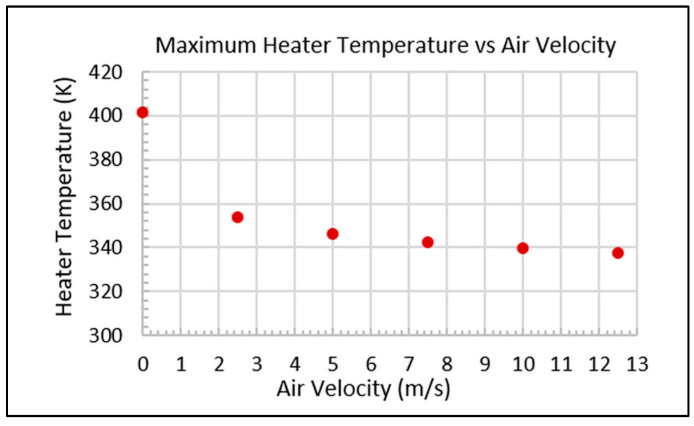
FEM convective cooling prediction for Design 1: maximum heater temperature as a function of air velocity (0–12.5 m/s).

Following validation, the model was employed to predict the convective cooling behaviour of Design 1 by imposing the analytically derived h values from Table 2 on the top surface of the heater for air velocities in the range 0–12.5 m/s. The simulated maximum heater temperature (T_h_) as a function of air velocity (U_air_) is presented in Figure 4. The results predict a monotonic decrease in heater temperature with increasing air velocity, indicating that the printed CNT-PLA element is, in principle, suitable for thermal anemometric operation. Furthermore, the maximum heater temperature (T_h_) is predicted to decrease from 401.42 K at zero flow to 329.01 K at 12.5 m/s, corresponding to a total convective cooling of 72.41 K, while for air velocities above 5 m/s, the thermal response exhibits reduced sensitivity. We should note that the specific results are presented as an initial numerical assessment that motivates the subsequent design evolution, rather than as an experimentally validated flow-sensing result.

## 4. Design 2: Enhanced Flat Heater—Thermal Localisation and Power Efficiency

### 4.1. Geometry and Design Rationale

**Figure 5:**
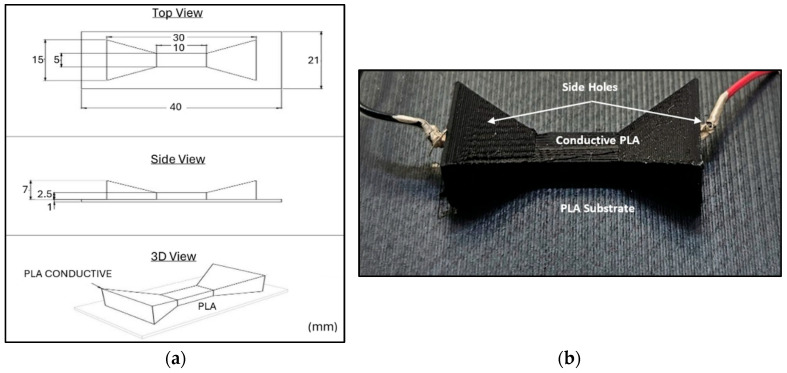
Design 2—Enhanced flat heater: (**a**) engineering drawing (top, side, and 3D views); (**b**) photograph of the fabricated device; side holes are incorporated at the contact pad locations to permit electrical connection via conductive adhesive.

In Design 1, the relatively uniform conductive cross-section distributes Joule heating broadly along the element, limiting temperature localisation in the sensing region. In order to improve thermal sensitivity and electrothermal efficiency, a second device generation was designed featuring a significantly more pronounced hourglass geometry; the specific design evolution is enabled directly by the freedom of geometry available through the FDM process, which allows complex conductive trace shapes to be implemented in a single print step without additional tooling or process changes—a key advantage already demonstrated in [15,16] for different sensing geometries. In Design 2, the central conductive bridge retains the same 5 mm width as Design 1, while the conductive element height is reduced to 2.5 mm; consequently, the central electrical cross-section is reduced from 25 mm^2^ to approximately 12.5 mm^2^, while the overall footprint and substrate dimensions remain identical (40 × 21 mm); note that appropriate side holes are incorporated at the contact pad locations to permit electrical connection via conductive adhesive, improving contact reliability compared to the surface-applied adhesive interface of Design 1. The reduced-cross-section central region acts as a high-resistance constriction, concentrating current density and Joule heating in the sensing zone while the terminal pad regions remain comparatively cooler. The engineering drawing and fabricated device are presented in Figure 5.

### 4.2. Comparative Thermal Characterisation

**Figure 6:**
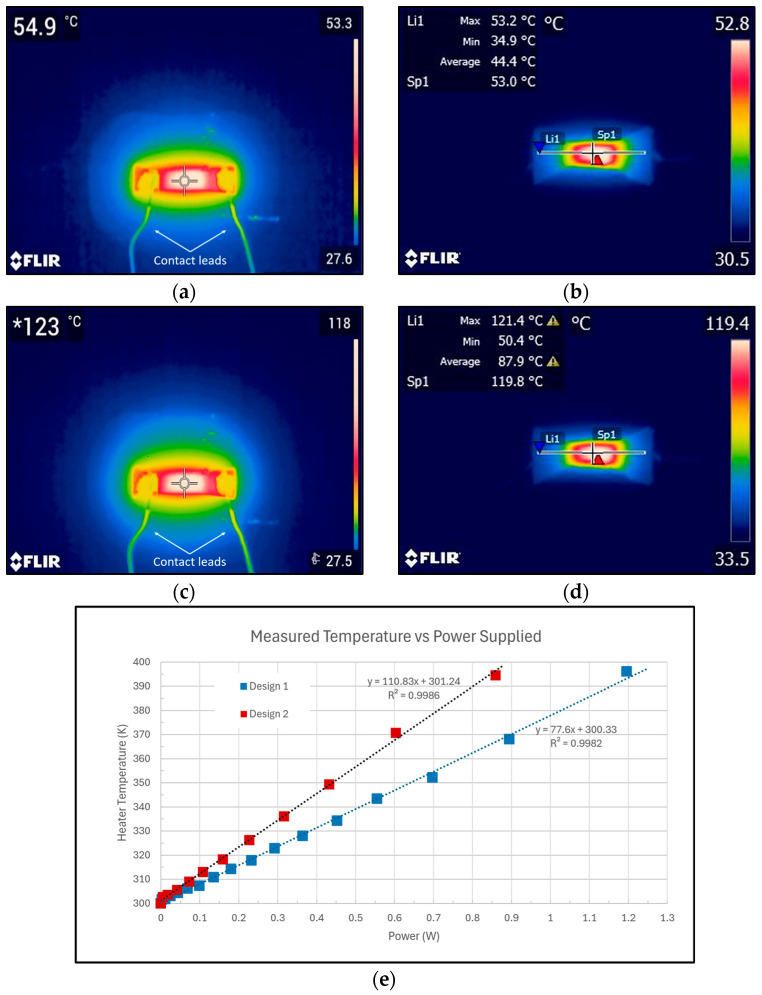
Comparative characterisation of Designs 1 and 2: IR thermal imaging of (**a**) Design 1 at 55 mA, (**b**) Design 2 at 35 mA, (**c**) Design 1 at 80 mA, (**d**) Design 2 at 55 mA; (**e**) measured heater temperature versus supplied power for both designs across their common operating range, from the stepped constant-current measurements.

Both Design 1 and Design 2 were examined under constant-current operation at current levels chosen to yield comparable peak temperatures, enabling a direct comparison of thermal distribution and temperature localisation; the corresponding IR thermal images at representative current levels are presented in Figure 6. As we can observe in the specific figure, in Design 1, the temperature distribution is relatively broad and uniform across the conductive element, with a gradient toward the contact pads, which remain significantly warmer than the surroundings; in Design 2, the distribution is sharply peaked at the reduced-cross-section central bridge, with the lateral terminal regions remaining noticeably cooler. In addition, no pronounced heating of the electrical leads or contact regions is observed in Design 2, indicating improved contact behaviour with reduced contact-related Joule heating and more effective power dissipation within the intended central constriction. The specific improvement in localisation directly translates to a higher signal-to-noise ratio for temperature sensing and as presented in the following section, to a substantially more efficient use of the supplied power.

### 4.3. Power Efficiency Comparison

The above results show that the geometric modification introduced in Design 2 achieves a significant improvement in electrothermal efficiency. In more detail, the presented experimental data (Figure 6) confirm that Design 2 reaches an equivalent maximum temperature to Design 1 at a reduced supplied power across the whole operating range, which is equal to approximately 26% at the highest temperatures examined and larger at lower power levels. Operating at a current of 55 mA, for example, Design 1 dissipates a power of 364 mW (R_1_ ≈ 120 Ω), reaching a peak temperature of 54.9 °C, whereas Design 2 achieves an almost identical peak temperature (53.2 °C) operating at 35 mA at a power of 227 mW (R_2_ ≈ 185 Ω), i.e., a reduction of approximately 38%. Accordingly, when Design 1 operates at 80 mA, with a corresponding power dissipation of approximately 1196 mW (its resistance having increased to R_1_′ ≈ 187 Ω), it reaches a peak temperature of 123 °C, while Design 2 achieves approximately the same peak temperature (121 °C) operating at 55 mA at a power of 874 mW (R_2_′ ≈ 289 Ω), which is reduced by approximately 26% with respect to the Design 1 case.

The complete temperature–power characteristics of both designs are compared in Figure 6e, obtained from the stepped constant-current measurements of Section 2.3. As we can see, both responses are essentially linear (Design 1 slope is equal to 77.6 K/W—coinciding with the thermal resistance Rth of Section 3.3, while Design 2 slope is equal to 110.8 K/W), and Design 2 lies consistently above Design 1 in the T–P characteristic curve, i.e., it reaches a given peak temperature at a consistently lower supplied power throughout the common operating range, confirming that the efficiency gain is maintained across the range rather than occurring only at the two representative points mentioned in the previous paragraph. We should note that the highest-power Design 1 measurement point lies above its projected linear fit, i.e., Design 1 reaches its maximum temperature (123 °C at 80 mA) at slightly lower power than the trend predicts; thus, the reported reduction of approximately 26% is conservative since the projected linear fits imply a reduction of approximately 30%.

The power-efficiency improvement is attributed to the geometric modification, which concentrates Joule dissipation within the reduced-cross-section sensing region and enables a higher local temperature rise for a given supplied power. This qualitative interpretation can be made more explicit by distinguishing the electrical redistribution of Joule heating from the subsequent thermal localisation. Electrically, the reduction in the central cross-section from 25 mm^2^ in Design 1 to 12.5 mm^2^ in Design 2 increases the resistance per unit length of the central region by a factor of two, assuming the same material resistivity; consequently, under constant-current excitation, a larger fraction of the total Joule power is dissipated in the intended sensing zone. Thermally, the reduced-cross-section centre lowers the axial thermal conductance of the conductive path and therefore limits heat spreading from the heated centre towards the wider terminal arms; as a result, the Joule power dissipated in this region produces a larger local temperature rise and a more sharply localised temperature maximum. In addition, the positive TCR of the CNT-PLA introduces a secondary electrothermal feedback, since the locally heated region becomes more resistive and therefore promotes further local Joule dissipation under constant-current operation. Since the material and printing parameters remain unchanged, the observed efficiency gain is attributed to the geometric modification; thus, a systematic FEM-based parametric assessment of the relative roles of electrical redistribution and thermal localisation, through independent variation in the central-region cross-sectional dimensions, would constitute a natural extension of the present analysis.

## 5. Design 3: Monolithic Ring-Type In-Pipe Airflow Sensor

### 5.1. Device Concept and Geometry

The insights gained from Designs 1 and 2 motivated the development of a fully monolithic, pipe-embedded thermal airflow sensor (Design 3), realised as a conductive ring co-printed as an integral segment of the pipe wall and hereafter referred to as the ring-type sensor. The sensing concept is directly analogous to the hot-wire anemometer principle (detailed in Section 5.2), but its geometry is adapted for in-pipe deployment, enabling installation directly within the pipe structure without any secondary assembly operations. We should note that this approach is consistent with the broader objective, shared across the authors’ previous work [15,16,17], of developing sensing devices whose complete structure—including the sensing element, the mechanical support, and the electrical contact pads—is fabricated in a single 3D printing step; in the specific case, the ring-type sensor additionally forms an integral segment of the pipe itself, a feature not achieved in any of the previous device generations.

**Figure 7:**
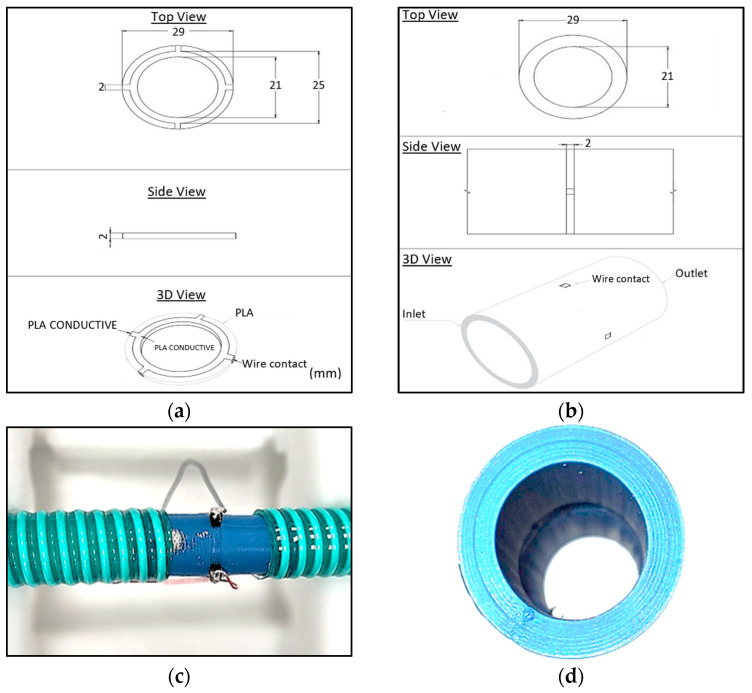
Design 3—Monolithic ring-type airflow sensor: (**a**) standalone sensor geometry showing ring dimensions and four wire contact positions at 90° intervals; (**b**) sensor integrated within a 3D-printed pipe segment; (**c**) photograph of the pipe-integrated prototype installed in the experimental flow setup; (**d**) photograph of the internal surface of the printed pipe segment, with image brightness adjusted to improve visibility of the inner bore surface.

In more detail, the sensor comprises a ring-shaped conductive CNT-PLA element with an outer diameter of 25 mm, inner bore of 21 mm, ring width of 2 mm, and axial thickness of 2 mm, co-printed within a non-conductive PLA annular disc. Four equidistant wire contact points at 90° intervals on the ring circumference divide the conductive element into four quarter-arc segments; the characterisation presented in the following sections was performed by exciting a single quarter-arc (the conductive path between two adjacent contact points) via the external source meter. Figure 7 presents the standalone ring-type sensor geometry and its integration into a 3D-printed pipe segment, together with photographs of the pipe-integrated prototype installed in the experimental flow setup and the internal surface of the printed pipe segment. The ring geometry ensures approximately symmetric exposure to the flow across the pipe bore, while the smooth inner bore surface avoids significant flow disturbance within the sensor. Note that the specific device is fabricated on the same desktop platform as in the previous design stages, confirming that the entire sensing device can be manufactured on-site, on demand, without reliance on any external manufacturing facilities.

### 5.2. Operating Principle

The ring-type sensor operates as a constant-current (CC) thermal anemometer. Its principle of operation is directly analogous to the hot-wire sensing principle discussed in [17], where a Joule-heated element is cooled by the incoming airflow, and the resulting change in its resistance provides the sensing signal. More generally, this operating principle belongs to the established family of thermal flow sensors, in which heat exchange between a heated element and the surrounding fluid is used to infer flow velocity or flow rate; such thermal anemometric methods have been widely applied to gas-flow and pipe-flow measurements [18,19], an area addressed by the proposed sensor. In the present case, at fixed excitation current I, Joule heating raises the temperature of the heated ring segment above ambient and establishes a no-flow steady-state resistance. When airflow is introduced, convective cooling reduces the temperature of the heated segment; since the CNT-PLA composite possesses a positive TCR, as established in Section 3.2, the resistance decreases accordingly. We should note that the heated element is a single quarter-arc of the ring, that is, the conductive segment between two adjacent contact points. Therefore, when the sensor is operating in CC mode, as in the characterisation performed in Section 5.3, increasing airflow velocity is expected to reduce the sensing element temperature and, consequently, the measured resistance. We should note that the intended measurand of the device is the mean air velocity of the steady flow through the sensor bore; for a fixed and calibrated bore cross-section, this can be related to the corresponding volumetric flow rate. The device is therefore not intended to resolve local point velocities, velocity profiles, or fast flow transients; the characterisation presented in Section 5.3 and the marine monitoring applications discussed in Section 6 should be interpreted accordingly. Furthermore, since the convective response depends on the specific bore geometry and inlet configuration, the derived transfer characteristic applies to the device as an integrated pipe fitting rather than to the ring element in isolation.

### 5.3. Flow Response Characterisation at Constant Current (40 mA)

**Figure 8:**
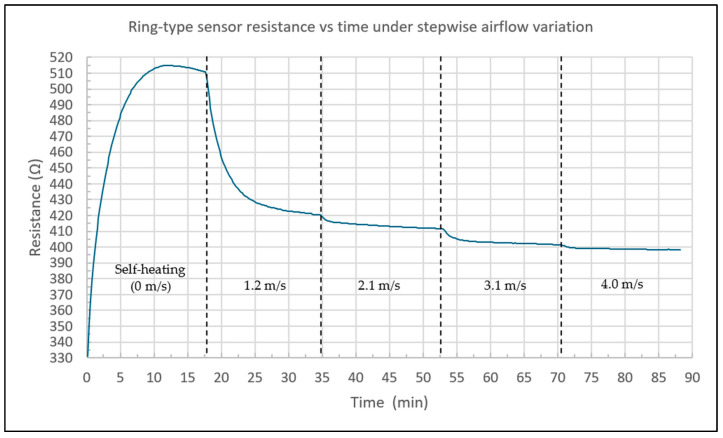
Ring-type sensor resistance time-series under constant-current excitation at 40 mA during stepwise airflow variation from zero flow to 4.0 m/s.

The ring-type sensor was characterised under airflow conditions at a constant operating current of I = 40 mA. The specific operating current was selected on the basis of the electrothermal characterisation of the preceding design stages: at 40 mA, the heated quarter-arc develops a no-flow temperature rise of approximately 72 K above ambient, providing a strong thermoresistive signal—in accordance with the observation in [17] that a higher operating temperature yields larger output variations for the same flow change—while the corresponding peak temperature remains within the range already characterised for Designs 1 and 2 (up to 123 °C, Section 4.3), where linear thermoresistive behaviour was established, and the required supplied power remains below 1 W. Note that the device was allowed to reach thermal steady state at each flow condition before recording the corresponding representative resistance value. The raw resistance time-series for the complete characterisation, comprising 450 measurements over approximately 88 min, is presented in Figure 8. In more detail, during the initial self-heating phase under no-flow conditions (0–18 min), the sensor resistance rises from a cold-start value of 331 Ω to a peak value of approximately 515 Ω. When airflow is introduced in the following successive velocity steps of 1.2, 2.1, 3.1, and 4.0 m/s, a progressive decline is observed in the resistance value, reaching 398 Ω at the maximum tested velocity; thus, a total resistance drop of ΔR ≈ 117 Ω is recorded over the 0–4 m/s range, corresponding to approximately 22.7% of the heated no-flow baseline. We should note that the response sensitivity is highest at low velocities, in accordance with the convective heat transfer characteristics predicted by Equations (5)–(7) and supported by the FEM analysis presented in Section 5.5. At higher velocities, the response flattens progressively: the step from 3.1 to 4.0 m/s produces a resistance change of only 3.5 Ω, corresponding to a temperature change of about 1.4 K based on the deduced TCR (see Section 3.2). This indicates that incremental convective cooling has become very small, since the remaining temperature difference between the heated ring and the incoming airflow is no longer sufficient to produce significant additional convective cooling; thus, the sensor response approaches a plateau. We should note that the specific step nevertheless remains clearly resolvable: the sample standard deviation of the steady-state resistance signal over the final forty samples of each velocity plateau, corresponding to approximately the final eight minutes of each plateau, was 1.1, 1.6, 0.5, 0.4, and 0.1 Ω at 0, 1.2, 2.1, 3.1, and 4.0 m/s, respectively. These values remain below 0.4% of the corresponding plateau resistance level in all cases; therefore, even the small resistance step between the two highest velocities exceeds the corresponding plateau scatter by approximately one order of magnitude. Furthermore, the characterisation was not extended beyond 4.0 m/s since, at the specific operating current, the response has already entered its asymptotic flattening regime, as noted above and quantified below; thus, higher velocity steps would provide limited additional information. Operation at a higher excitation current would extend the usable span towards higher velocities, in accordance with the higher-temperature operation observation in [17].

In order to provide a quantitative transfer characteristic for the device, the steady-state response under stepwise airflow variation was further analysed through the heat balance governing constant-current operation. In more detail, at each velocity plateau, the sensor was allowed to reach thermal equilibrium; therefore, the corresponding resistance (R), supplied power (P), and temperature rise (ΔT) can be treated as steady-state values for the specific plateau. Thus, the electrical power dissipated in the heated element is balanced by the heat removed through the surrounding thermal paths. This balance can be expressed as $P=G_{eff}\left(U\right)\cdot \Delta T$, where $G_{eff}\left(U\right)$ is the velocity-dependent effective thermal conductance of the heated ring–pipe–flow system and ΔT is the temperature rise in the heated element above ambient. In classical constant-temperature hot-wire anemometry, King’s law is commonly written as:

(15) $$E^{2}=A_{C}+B_{C}\cdot U^{n}$$

where E is the voltage across the heated element, $A_{C}$ and $B_{C}$ are calibration constants and n is the calibration exponent. Since $E^{2}=P\cdot R_{w}$, where P is the electrical power dissipated in the heated element and $R_{w}$ is its resistance at the operating temperature, under constant-temperature operation, ΔT and $R_{w}$ are maintained fixed; therefore, Equation (15) may be rewritten as:

(16) $$G_{eff}\left(U\right)=A+B\cdot U^{n}$$

with $A=A_{C}/\left(R_{w}\Delta T\right)$ and $B=B_{C}/\left(R_{w}\Delta T\right)$. Therefore, the King-law velocity dependence can equivalently be interpreted as a power-law dependence of the effective thermal conductance. We should note that in the present sensor, ΔT is not held constant across the examined velocities, since the device is operated under constant-current rather than constant-temperature conditions; however, each velocity plateau provides a well-defined steady-state temperature rise. Thus, the effective thermal conductance of the printed ring–pipe–flow system was evaluated for each plateau point of the stepwise characterisation as $G_{eff}\left(U\right)=P/\Delta T$, where $P=1000\cdot I^{2}\cdot R$ is the supplied Joule power in mW and $\Delta T=\left(R-R_{ref}\right)/\left(\alpha \cdot R_{ref}\right)$ is estimated from the measured plateau mean resistance employing the deduced TCR from Section 3.2. Adopting the classical square-root exponent of King’s law (*n* = 0.5), the constants A and B in Equation (16) can therefore be derived as follows: A represents the zero-flow effective thermal conductance, i.e., A = G_eff_ (0) = 11.163 mW/K. The values of B for the four non-zero velocity plateaus can be calculated by rearranging Equation (16), i.e., $B_{i}=\left(G_{i}-A\right)/\sqrt{U_{i}}$ resulting in 6.490, 6.173, 6.420, and 6.128 $mW/\left(K\cdot {\left(m/s\right)}^{0.5}\right)$ respectively, yielding a mean value of $B=6.303\;mW/\left(K\cdot {\left(m/s\right)}^{0.5}\right)$. Thus, the resulting fitted King-type effective-conductance characteristic is:

(17) $$G_{fit}\left(U\right)=11.163+6.303\sqrt{U}$$

In order to compare this conductance-based expression directly with the measured sensor output, Equation (17) was transformed back into the resistance domain by combining $P=1000\cdot I^{2}\cdot R$ with $\Delta T=\left(R-R_{ref}\right)/\left(\alpha \cdot R_{ref}\right)$, resulting in:

(18) $$R_{pred}\left(U\right)=\frac{G_{fit}\left(U\right){\cdot R}_{ref}}{G_{fit}\left(U\right)-1000\cdot I^{2}\cdot \alpha \cdot R_{ref}}$$

**Figure 9:**
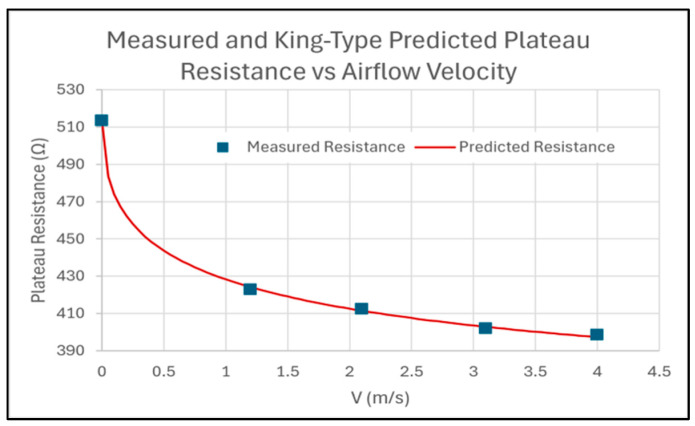
Measured steady-state plateau resistance values of the ring-type sensor and corresponding King-type effective-conductance fit over the examined 0–4 m/s range.

The resulting predicted resistance curve is compared with the measured steady-state plateau resistances in Figure 9. The King-type prediction reproduces the measured plateau resistances with $R^{2}=0.9995$, and a maximum absolute deviation of approximately 1.3 Ω. Therefore, within the examined 0–4 m/s range, the steady-state response is well described by a King-type square-root velocity dependence. We should note that the fitted constant A represents the combined zero-flow conductance of the assembly, including conduction towards the printed pipe structure, natural convection, and other background losses, while the term $B\sqrt{U}$ captures the forced-convection contribution. The corresponding local sensitivity dR/dU, calculated as the finite-difference slope between successive steady-state plateau mean resistances, decreases in magnitude from 76 Ω/(m/s) over the 0–1.2 m/s interval to 11.6, 10.4, and 3.9 Ω/(m/s) over the subsequent intervals, quantifying the progressive flattening of the R–U characteristic. The mean full-range sensitivity is approximately 29 Ω/(m/s).

An estimate of the noise-limited detection capability—the smallest air velocity expected to be distinguishable from the no-flow baseline—can be obtained from the requirement that the flow-induced resistance change exceed twice the zero-flow scatter. In more detail, for a value of σ ≈ 1.1 Ω (corresponding to the resistance spread at the zero velocity plateau) and the lowest-velocity (1.2 m/s) sensitivity dR/dU ≈ 76 Ω/(m/s), U_min_ ≈ 2σ/|dR/dU| = 2 × 1.1/76 ≈ 0.03 m/s. We should note that the specific figure constitutes an extrapolated, noise-limited estimate and not an experimentally demonstrated detection limit, since the lowest non-zero velocity tested is 1.2 m/s and the employed sensitivity corresponds to the average rate of change in the resistance over the 0–1.2 m/s interval; its experimental verification is included in the finer low-velocity testing identified in the future work of Section 7. Also, the deduced velocity resolution degrades progressively towards higher velocities as the local sensitivity decreases (to approximately 3.9 Ω/(m/s) over the 3.1–4.0 m/s interval).

The extrapolated minimum detectable velocity of approximately 0.03 m/s can be placed in context against the corresponding values reported for other typical flow-sensing devices (reported in the literature or commercially available) as follows. Silicon micromachined thermal flow sensors have experimentally demonstrated minimum detectable air velocities of the order of 0.001 m/s, with the superimposed natural convection—rather than the electrical noise floor—identified as the practical lower bound [29], while commercially available thermal anemometry transducers intended for low-velocity applications specify lower range limits of 0.05 m/s [30]. Among fully 3D-printed airflow sensors, considerably higher minimum detectable velocities have been reported, ranging from approximately 0.06 m/s for printed conducting-polymer micro-hair structures [31] to approximately 1.2 m/s for an FDM-printed piezoresistive device [32]. From the above, we can safely assume that the present device, subject to the experimental verification discussed above, is positioned between the micromachined thermal flow sensors and the fully printed devices reported to date, at a level comparable to the lower range limit of commercial thermal anemometry instrumentation.

### 5.4. Flow Switching and Signal Reversibility

**Figure 10:**
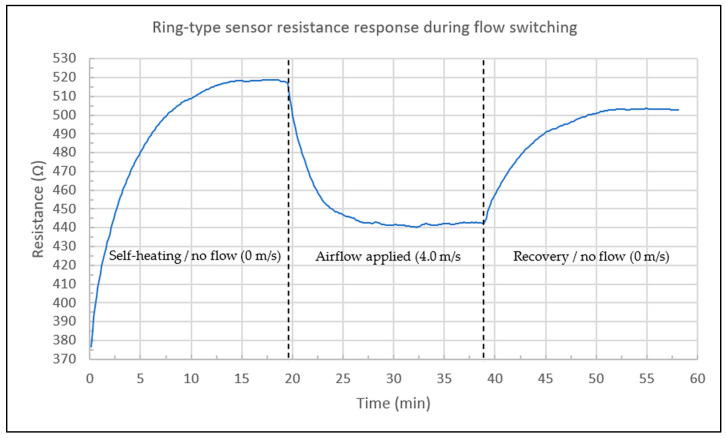
Ring-type sensor resistance response under constant-current excitation at 40 mA during flow switching between zero-flow and 4.0 m/s airflow conditions.

Following the successful characterisation of the device as a constant-current thermal anemometer under steady airflow conditions, a flow switching experiment employing a constant current of 40 mA was conducted in order to assess the reversibility of the sensing response; the recorded resistance response during the no-flow, airflow, and recovery stages is presented in Figure 10. As shown in the figure, after the airflow was interrupted, the ring resistance rises from the flow-cooled value of approximately 441 Ω toward a recovered level of approximately 503 Ω over approximately 13 min, remaining approximately 16 Ω below the initial no-flow value of approximately 519 Ω. The recovery curve follows an exponential-like trend, qualitatively consistent with a first-order thermal recovery process governed by the effective thermal mass of the ring element and its thermal coupling to the surrounding pipe structure. We should note that the recovered resistance does not fully return to the initial recorded no-flow value but stabilises approximately 16 Ω below it, a margin that corresponds to about 3% of the heated baseline. In more detail, approximately 79% of the cooling-induced resistance change is recovered once the airflow is removed, while the remaining approximately 21% persists as an unrecovered offset after the single examined cycle; the residual offset may be attributed either to a small, partly irreversible change in the conductive network upon thermal cycling, consistent with the sintering sensitivity reported for the same filament in [16], or to slightly altered no-flow convective conditions after the flow cycle; the present single switching record does not allow these two mechanisms to be distinguished. We should note that the reported recovery ratio and residual offset are derived from a single interruption–recovery cycle; establishing cycle-to-cycle repeatability and the long-term stability of the no-flow baseline requires the repeated switching experiments identified in the future work of Section 7.

### 5.5. FEM Modelling of the Ring-Type Sensor Flow Response

A finite-element model of the ring-type sensor was developed in COMSOL Multiphysics^®^ to reproduce the measured flow response based on the analytically derived convective boundary conditions, presented in Table 2. Beyond reproducing the experimentally observed resistance variation, the model provides access to quantities that are not directly measurable during flow characterisation, such as the spatial temperature distribution along the heated quarter-arc and the relative contribution of the surrounding pipe structure to heat dissipation. The comparison between the simulated and experimentally estimated temperature rise, obtained from resistance measurements using the TCR deduced in Section 3.2, also serves to assess the adequacy of the assumed heat-transfer coefficients and to interpret the residual deviations between model and experiment. Furthermore, the FEM framework can be employed as a predictive design tool for future optimisation of the ring geometry, reduction in thermal mass, adjustment of the heated arc length, and investigation of alternative sensor topologies with improved sensitivity and dynamic response.

In contrast to the Design 1 FEM, where the experimentally measured power was applied directly as a volumetric heat source, the Design 3 model employs a fully coupled electrothermal simulation; that is, a constant current of 40 mA is applied to one quarter-arc element of the ring-type sensor, while the electrical resistivity of the CNT-PLA domain is modelled as temperature-dependent according to Equation (1). The Joule heat generation is then calculated from the coupled electric-current solution, and the resulting temperature field and electrical resistance are solved simultaneously. Convective cooling was imposed on the model boundaries through surface heat transfer coefficients: a value of h = 8 W/(m^2^·K) was assigned to all external surfaces exposed to the open laboratory environment, while an effective value of h = 100 W/(m^2^·K) was applied locally to the side faces of each active contact pad in order to account for the conductive heat sinking through the connecting wires. For the internal surfaces of the pipe segment, which are in direct contact with the airflow, a value of h = 3 W/(m^2^·K) was assigned under zero-flow conditions, while velocity-dependent convective heat transfer coefficients, corrected as discussed below, were applied for the forced-flow cases.

The analytically derived convective heat transfer coefficients employed for Design 3 should be regarded as first-order estimates. This is because the ring-type sensor is directly fitted inside a larger upstream tube, as shown in Figure 7c, and the airflow entering the 21 mm sensor bore from the 29 mm upstream tube undergoes an abrupt contraction, with an area ratio of approximately 0.52. The resulting local acceleration through the contraction, together with downstream recirculation regions, is expected to enhance the local convective heat transfer coefficient above the values predicted by the fully developed smooth-tube correlations and reported in Table 2. This interpretation is consistent with the findings of Ghajar and Tam [33], who showed that in circular tubes, the heat transfer coefficient and the transition Reynolds-number range depend strongly on the inlet disturbance configuration. Therefore, at zero airflow velocity, natural convection conditions apply on both the internal and external surfaces of the sensor, and no correction is necessary; the internal surface accordingly retains its baseline coefficient of h = 3 W/(m^2^·K), representative of free convection of air within the pipe. For the forced-flow cases, however, an effective correction factor of 3.5 was calibrated against the experimental data, consistent with the enhanced convective heat transfer expected downstream of an abrupt contraction. The fact that a single correction factor provides comparable agreement across all the tested flow velocities supports the interpretation that the enhancement arises primarily from the fixed geometric contraction rather than from a velocity-dependent mechanism. Consequently, the FEM model of the forced-flow cases should be regarded as partly fitted rather than fully predictive: the functional form of the velocity dependence follows the analytical correlations of Section 2.4, while its absolute level is calibrated against the experimental data through the specific factor. The sensitivity of the predicted response to the adopted heat-transfer coefficients can be calculated directly from the measured effective conductances of Section 5.3, independently of the FEM solution: the forced-convection term B·U^0^.^5^ accounts for approximately 38% to 53% of the total effective thermal conductance between 1.2 and 4.0 m/s; therefore, a ±20% perturbation of the forced-convection coefficient translates into a change of approximately 8–11% in the predicted steady-state temperature rise, comparable to the expanded uncertainty of the experimentally estimated ΔT values (approximately 11% at k = 2). At zero flow, the natural-convection coefficients contribute only a minor share of the total effective conductance, which is dominated by conduction into the printed pipe structure; this is consistent with the close zero-flow agreement of approximately 3% despite the inherently larger uncertainty of natural-convection estimates. The role of the omitted surface-radiation term is discussed in Section 3.4; for the ring-type sensor, its effect is bounded by the same sensitivity argument, since any radiative contribution at the surfaces is equivalent to a moderate increase in the corresponding fixed h values, which for the forced-flow cases is absorbed by the calibrated correction factor.

The simulated steady-state temperature rise in the active quarter-arc above ambient, ΔT, is compared against the experimentally derived values in Table 3 across the full range of tested airflow velocities. At zero flow, the model reproduces the experimental temperature rise to within approximately 3%, while for the flow cases above 1.2 m/s, the agreement lies within approximately 5–8%; at the lowest velocity step (1.2 m/s), the simulation overpredicts the temperature rise by 20%. This remaining noticeable deviation at 1.2 m/s is attributed to the simplified assumption of a constant laminar Nusselt number (Nu = 4.36), despite the disturbed flow conditions at the sensor inlet, which are expected to produce entrance-region effects not captured by the fully developed constant-Nu assumption. We should note that the experimental ΔT values were obtained from the measured resistance variation using the TCR value deduced in Section 3.2, whereas the simulated values correspond to the arc temperature rise extracted from the coupled electrothermal solution. Applying the uncertainty procedure described in Section 2.5, the experimentally estimated ΔT values carry a relative standard uncertainty dominated by the uncertainty of the deduced TCR and the plateau-resistance scatter. Since the largest plateau-scatter contribution is about 1.7% and the TCR contribution is 5%, combining them in quadrature results in a relative standard uncertainty of 5.3% for the experimentally estimated ΔT; therefore, the corresponding expanded uncertainty is approximately 11% at a coverage factor k = 2, which is used below as a practical uncertainty envelope for assessing the FEM–experiment deviations. The airflow-velocity uncertainty, estimated in Section 2.5 from the UNI-T UT363 specification as approximately ±0.08 to ±0.21 m/s over the examined range, affects the assigned velocity values and therefore the horizontal positioning of the FEM–experiment comparison points, but it is not combined with the vertical uncertainty of the experimentally estimated ΔT values.

Across the five examined conditions, the mean absolute deviation between the simulated and the experimentally estimated temperature rise is 8.4%, reducing to 5.5% when the 1.2 m/s laminar case is excluded; thus, the deviations for the zero-flow condition and for the velocities above 2 m/s are comparable to the standard uncertainty of the experimentally estimated ΔT and lie within the corresponding expanded uncertainty envelope (approximately 11% at a coverage factor k = 2). Consequently, the model should be regarded as a calibrated, semi-empirical representation of the sensor response rather than as independently validated. In more detail, for the forced-flow cases, where the internal heat-transfer level is scaled through the correction factor derived from the same experimental data set, the observed agreement demonstrates the internal consistency of the adopted description, while the zero-flow condition—where no correction factor is applied—constitutes the only independently predicted operating point, reproduced within approximately 3%. Note that the fully developed laminar assumption at the lowest tested velocity marks the boundary of applicability of the adopted correlations.

## 6. Discussion

As a first and more comprehensive remark from the experimental results obtained, we should note that the proposed device’s proof-of-concept has been validated; the CNT-PLA composite employed exhibits a consistent thermoresistive response within the performed single-prototype characterisation, with the derived effective device-level parameters (TCR = 0.0075 K^−1^, ρ_0_ = 0.204 Ω·m), in line with the pronounced thermoresistive sensitivity reported for the same filament in [16], obtained on an independently printed device of different geometry. The sign and magnitude of TCR in CNT/polymer composites are known to be sensitive to filler concentration, matrix properties, and processing conditions, with both positive and negative values reported at comparable loadings [26,27]; the positive TCR observed here—resistance increases with temperature and therefore decreases upon convective cooling—is the key parameter governing the ring-type sensor response. In more detail, all three quantitative targets set in Section 1 have been met: the ring-type sensor exhibits a monotonic response with a total resistance variation of approximately 22.7% of the heated no-flow baseline (target: at least 10%); Design 2 achieves a power reduction of approximately 26% at the highest temperatures examined through geometry alone (target: at least 20%); and the FEM model reproduces the experimental temperature rise within 3.1% at zero flow and within 5–8% for the forced-flow cases above the laminar regime (target: approximately 10%), the sole exception being the 1.2 m/s laminar case, as discussed in Section 5.5. We should note that, for the forced-flow cases, the specific agreement is obtained after calibrating the internal convective level through the correction factor of Section 5.5, whereas the zero-flow prediction involves no correction factor.

Regarding the Design 1 FEM model, the calibrated COMSOL simulation accurately predicts the T–P relationship at moderate power levels, as presented in Figure 3b. At higher power, though, the simulation slightly overestimates the maximum temperature recorded on the heating element (T_h_); the specific deviation is attributed primarily to simplified boundary conditions and constant-property assumptions, which do not fully capture parasitic heat losses or the non-uniform thermal response of the printed CNT-PLA/PLA structure arising from its 3D-printed morphology. We should note that the use of analytically derived h values as FEM boundary conditions, rather than a full CFD solver, is consistent with the simplified heat transfer analysis employed in [17], where the same empirical equation was employed to establish the expected saturation behaviour of the hot-wire sensing element.

The transition from Design 1 to Design 2 demonstrates that FDM geometry alone is a sufficient parameter for electrothermal optimisation. The approximately 26% reduction in required power for an equivalent temperature rise at the highest temperatures examined was achieved solely through narrowing the central conductive trace, without any change in material, printing parameters, or post-processing. Note that the above principle is of direct practical relevance for marine sensing applications, where battery-operated or remotely powered sensing systems are subject to strict power budget constraints.

As already demonstrated in [15,16,17], the same commercially available desktop printer and filament are sufficient for fabricating the complete sensing device on-site, on demand, addressing the operational constraints of Section 10.3 of the ISM Code [13] regarding safety-critical spare parts. We should note that the flat-heater sensing geometry demonstrated in Designs 1 and 2 can be directly embedded into any 3D-printed structural component (e.g., a bracket or housing), enabling simultaneous thermal monitoring and structural functionality within a single printed part—a capability that is of direct relevance for condition-based maintenance applications in the marine sector.

Regarding the repeatedly invoked low-cost character of the approach, a first-order techno-economic estimate can be provided. The complete pipe-integrated Design 3 prototype has a mass of approximately 36 g, of which only about 2 g corresponds to the conductive CNT-PLA element and contact pads; at current indicative supplier prices of approximately €70/kg for the conductive filament and €20/kg for standard PLA, the raw material cost per device is approximately €0.8. Adding the electrical energy of a single desktop print run of approximately 4 h (of the order of 0.5 kWh, i.e., approximately €0.15) yields a raw consumables cost of the order of €1 per device; the specific figure excludes the amortisation of the printer itself (a desktop unit shared across all sensing devices of the present family [15,16,17]), labour, quality-assurance testing, and the readout electronics, and should therefore not be interpreted as a complete device cost. Even so, the estimated consumables cost lies two to three orders of magnitude below the typical acquisition cost of industrial thermal flow-monitoring instruments; furthermore, in the marine context, the avoided logistics and stock-keeping costs discussed in Section 1 [13,14] can exceed the acquisition cost itself, particularly for vessels operating in remote locations.

At the same time, an objective assessment of the proposed on-site manufacturing approach requires that its limitations are equally acknowledged. FDM printing in a shipboard environment is subject to conditions that differ substantially from those of a laboratory: ambient temperature variations and high humidity affect the hygroscopic PLA feedstock and the inter-layer adhesion, while vibrations transmitted from the propulsion machinery can degrade dimensional accuracy during printing. In addition, the electrical characteristics of CNT-based conductive filaments are known to be sensitive to filler concentration and processing conditions [26,27], so a degree of variability between filament batches and between printer platforms should be expected; the post-printing thermal treatment discussed in [16] mitigates part of the specific variability by stabilising the conductive network, yet device-to-device statistics across printers and material batches remain to be established. Equally, the marine service environment itself—sustained high humidity, salt-laden atmospheres, and temperature cycling—has not been represented in the present characterisation, which was performed under laboratory conditions; environmental exposure testing of printed CNT-PLA sensing elements therefore remains a prerequisite for shipboard deployment. Consequently, the claimed advantages are expected to hold when printing takes place in a reasonably conditioned space—such as an onboard workshop—with dry-stored filament, combined with a simple post-fabrication electrical verification of the printed element prior to installation; under harsher conditions, fabrication at the nearest port facility remains a viable alternative that still avoids the logistics constraints discussed in Section 1.

Concerning the ring-type sensor (Design 3), the observed ΔR ≈ 117 Ω over 0–4 m/s at 40 mA corresponds to a mean sensitivity of approximately 29 Ω/(m/s); higher sensitivity at low velocities is in accordance with the convective heat transfer characteristics predicted by Equations (5)–(7). We should note that higher operating currents yield larger absolute ΔR for the same flow change, as reported in [17], confirming that excitation current is an effective sensitivity parameter. Furthermore, the thermal equilibration times observed (several minutes per flow step) reflect the relatively large thermal mass of the printed ring and pipe structure; the specific response time is compatible with steady-state flow monitoring applications such as mean pipe flow measurement and ventilation rate monitoring, where measurement update rates of minutes are operationally acceptable. Representative marine use cases of this type include the periodic verification of ventilation and air-supply lines and the condition monitoring of low-criticality pneumatic ducts; applications requiring faster dynamic response, such as engine intake transient measurement or fast leakage alarms, fall outside the intended scope of the present device and would require the reduced thermal mass identified in the future work. We should also note that in the flow switching experiment approximately 79% of the cooling-induced resistance change was recovered once the airflow was removed, the remaining approximately 21% persisting as a residual offset of about 3% of the heated baseline; the specific offset is well within the tolerance of the steady-state monitoring applications targeted here, and is consistent either with a minor, partly irreversible change in the conductive network upon thermal cycling, as reported for the same filament in [16], or with slightly altered no-flow convective conditions after the flow cycle. Such partial settling of the conductive network on first thermal cycling is expected to be a saturating effect, so the no-flow baseline should stabilise after the initial cycles rather than drift progressively; its systematic verification is left for future work. Furthermore, the diminishing sensitivity at higher velocities is inherent to constant-current thermal anemometers: as the convective heat transfer coefficient h increases sublinearly with velocity, the incremental cooling per unit velocity progressively decreases, resulting in an asymptotic flattening of the R–U characteristic. This behaviour is captured quantitatively by the King-type effective-conductance fit derived in Section 5.3 (Equations (16)–(18)), which reproduces the measured plateau resistances with R^2^ = 0.9995 across the tested range. The element retains significant self-heating above ambient even at the highest tested velocity (approximately 27 °C above cold-start at 4 m/s), indicating that the sensor has not reached thermal equilibrium with the environment but rather that the differential sensitivity dR/dU has diminished below a practical threshold.

A comparison with classical hot-wire and hot-film anemometers [18] further clarifies the operating point of the proposed device. Conventional hot-wire and hot-film probes employ micrometre-scale metallic wires or thin metallic films, with TCR values typically of the order of 0.0036–0.0045 K^−1^ and very low thermal mass; as a result, they provide millisecond-scale response suitable for turbulence-resolved measurements, at the cost of mechanical fragility, dedicated bridge electronics, and considerably higher acquisition cost. The printed ring-type sensor, in contrast, combines a higher TCR (0.0075 K^−1^) with a substantially larger heated volume and stronger thermal coupling to the surrounding printed pipe structure; these features support a robust, distributed in-pipe sensing device but necessarily shift the device away from fast-response anemometry and towards steady-state flow monitoring. Therefore, the ring-type sensor is not intended to compete with classical hot-wire or hot-film probes in dynamic response; rather, it addresses a different operating point, namely rugged, low-cost, steady-state monitoring of mean pipe flow, where measurement update rates of minutes are operationally acceptable and where the monolithic construction reduces the fragility and assembly constraints of conventional probes. We should note that, despite operating under constant-current rather than constant-temperature excitation, the steady-state response of the printed heating element was nonetheless shown in Section 5.3 to follow the classical King-type square-root velocity dependence (Equations (16)–(18)), indicating that the underlying convective heat-transfer physics remains consistent with established thermal anemometry theory despite the substantially different thermal mass and operating regime.

For Design 3, the coupled electrothermal FEM analysis provides a more appropriate assessment route than local IR thermography, since the active region is a curved quarter-arc embedded within the pipe structure and its spatially averaged temperature is not directly accessible during flow operation. The model applies the experimentally derived TCR and reference resistivity to the CNT-PLA domain and solves the Joule heating and temperature-dependent resistance simultaneously under 40 mA constant-current excitation. After accounting for the enhanced internal convective cooling caused by the abrupt contraction from the upstream tube to the sensor bore through an effective correction factor, the FEM-predicted temperature rise agrees closely with the experimentally estimated values, which in turn supports the transferability of the Design 1 TCR to the ring geometry. In particular, the zero-flow case is reproduced within approximately 3%, while the forced-flow cases above 1.2 m/s lie within approximately 5–8%. The larger deviation at 1.2 m/s is consistent with the limitations of the fully developed laminar-flow assumption under disturbed inlet conditions. Therefore, the FEM model should be interpreted as an effective electrothermal representation of the sensor response and as a design-optimisation tool for future ring geometries, rather than as a fully CFD-resolved flow model.

It is worth noting that all the elements comprising Design 3—the ring sensing element, the annular non-conductive support disc, the pipe segment, and the electrical contact pads—are printed through additive manufacturing in a single step; furthermore, direct communication to the necessary readout circuitry can be easily achieved through standard conductive adhesive on the integrated contact pads, with expected benefits in device complexity, process time, and cost, while avoiding fragile wire-bonded interfaces. The developed prototype can therefore be regarded as a stimuli-responsive printed device: the ring element changes its resistance state in response to the presence and magnitude of the incoming flow. We should note that, in contrast to the 4D-printing concept identified in [15], no shape transformation is involved; the connection is limited to the broader notion of a printed structure whose functional state responds to an external stimulus.

## 7. Conclusions

In the present study, the design evolution of an additively manufactured ring-type thermal sensor for in-pipe flow monitoring is presented, exclusively employing FDM additive manufacturing technology. Three progressive design stages were investigated, fabricated, and characterised.

The initial flat heater (Design 1) was fully characterised, yielding a TCR of 0.0075 K^−1^ and a Thermal Resistance of 78 K/W, consistent with the pronounced thermoresistive sensitivity reported for the same filament in [16]. The calibrated COMSOL FEM model predicts the Joule heating behaviour in close agreement with the experimental data and indicates a convective cooling effect of 72.4 K at 12.5 m/s, supporting the device’s potential for thermal anemometry.

The enhanced flat heater (Design 2) achieved equivalent thermal localisation to Design 1 at approximately 26% lower supplied power, realised solely through geometric modification of the central conductive region. We should note that no change in material, printing parameters, or post-processing was required.

The monolithic ring-type sensor (Design 3) exhibited a monotonically decreasing resistance response to increasing airflow at 40 mA constant-current excitation, with a total ΔR ≈ 117 Ω over the 0–4 m/s range. Flow switching experiments showed that the resistance recovered approximately 79% of the cooling-induced change when the airflow was removed; the remaining approximately 21% (≈16 Ω, i.e., about 3% of the heated baseline) persists as a residual offset after the single examined cycle. The coupled electrothermal FEM model of the heated quarter-arc further supported the experimental response, reproducing the zero-flow temperature rise within approximately 3% and the forced-flow cases above 1.2 m/s within approximately 5–8%, after accounting for the enhanced convective cooling associated with the inlet contraction. In more detail, the steady-state flow response was further reduced to a compact transfer characteristic by fitting a King-type effective thermal-conductance relation to the five velocity plateaus; adopting the classical square-root exponent, the fitted relation reproduces the measured plateau resistances with R^2^ = 0.9995 and a maximum deviation of approximately 1.3 Ω, providing a practical calibration expression for the device.

Beyond the measured sensing response, reliance on desktop FDM fabrication alone preserves the intended marine-maintenance advantage of the proposed approach: low-cost, on-demand fabrication of replacement or application-specific sensing elements, with potential to reduce the need for extensive onboard spare-part inventories, in line with the broader use of additive manufacturing for spare-parts applications [34].

It is worth noting that the proposed ring-type sensor and its preceding design stages share the same building material and fabrication platform as the vortex shedding flow sensor [15], the piezoresistive strain sensor [16], and the engine air intake MAF sensor [17] previously reported by the authors, establishing a growing family of 3D-printed sensing devices for marine applications. We should note that the results presented herein establish the feasibility of the proposed sensing concept at a proof-of-concept level, employing a single prototype per design stage, under laboratory conditions that did not include humidity, salt exposure, vibration, temperature cycling, or material-batch variability; the transition towards a calibrated, field-deployable instrument depends on the items identified below. Future work includes CFD-informed/FEM-based optimisation of the ring-type sensor geometry and calibrated flow testing over a wider velocity range—including finer resolution in the low-velocity region below 1 m/s—using a traceable reference. Furthermore, it should include the reduction in the ring thermal mass for improved dynamic response, repeated flow switching cycles to characterise the reproducibility and long-term stability of the no-flow baseline, and the fabrication and characterisation of multiple nominally identical prototypes to establish device-to-device statistics across printers and material batches. Finally, the development of a field-deployable 3D-printed pipe fitting for validation in a marine monitoring application and environmental exposure testing under humidity, salt-mist, vibration, and temperature-cycling conditions representative of the marine service environment are required before practical deployment can be claimed.

## Figures and Tables

**Table 1:** Material properties of the standard PLA filament and the CNT-enriched conductive PLA employed in the FEM models; justification of the adopted values is provided in Section 2.4.

Property	Standard PLA	CNT-Enriched PLA	Units
Thermal Conductivity (k)	0.26	4.5	W/(m·K)
Density (ρ)	1141	1240	kg/m^3^
Specific Heat Capacity (Cp)	1200	1200	J/(kg·K)
Reference Resistivity (ρ_0_)	1.78 × 10^8^	0.204	Ω·m
TCR (α)	—	0.0075	K^−1^
Reference Temperature (T_0_)	300		K

**Table 2:** Analytically derived convective heat transfer coefficients employed as FEM boundary conditions. For Design 1, the flat-plate laminar correlation Equation (2) was employed. For Design 3 (D = 21 mm), Nu = 4.36 (constant heat flux) was adopted for laminar flow (Re < 2300), and the Dittus–Boelter correlation Equation (6) for the transitional and turbulent regime (Re > 2300).

Air Velocity (m/s)	Design 1			Design 3		
	Re (–)	h (W/(m^2^·K))	Nu Number (–)	Re_pipe_ (–)	h (W/(m^2^·K))	Nu Number (–)
0 (natural convection)	—	8.00		—	3.00	—
1.2	—	—		1611.71	5.48	4.36
2.1	—	—		2820.49	14.51	11.55
2.5	799.46	88.37	16.75	—	—	—
3.1	—	—		4163.58	19.81	15.77
4.0	—	—		5372.36	24.29	19.34
5	1598.92	124.97	23.69	—	—	—
7.5	2398.37	153.06	29.01	—	—	—
10	3197.83	176.73	33.50	—	—	—
12.5	3997.29	197.59	37.45	—	—	—

**Table 3:** Comparison of the FEM-predicted and experimentally estimated steady-state temperature rise (ΔT above ambient) of the ring-type sensor (quarter-arc) operating at a constant current of 40 mA, together with the corrected internal-surface convective heat transfer coefficient imposed at each airflow velocity.

Air Velocity (m/s)	Corrected Internal h (W/(m^2^·K))	FEM-Predicted ΔT (°C)	Experimentally Estimated ΔT (°C)	Deviation from FEM-Predicted (%)
0	3.00	71.40	73.61	−3.09
1.20	19.18	46.31	37.02	+20.05
2.10	50.79	34.87	32.81	+5.91
3.10	69.34	31.12	28.63	+8.00
4.00	85.02	28.66	27.23	+5.00

## Data Availability

The data presented in this study are available on request from the corresponding author.

## Abbreviations

The following abbreviations are employed in this manuscript:

Abbreviation	Definition
AM	Additive Manufacturing
CC	Constant Current
CFD	Computational Fluid Dynamics
CNT	Carbon Nanotube
FDM	Fused Deposition Modelling
FEM	Finite Element Method
IC	Internal Combustion
IR	Infrared
ISM	International Safety Management
MAF	Mass Air Flow
PLA	Polylactic Acid
TCR	Temperature Coefficient of Resistance
